# Colonic Epithelial PHLPP2 Deficiency Promotes Colonic Epithelial Pyroptosis by Activating the NF-*κ*B Signaling Pathway

**DOI:** 10.1155/2021/5570731

**Published:** 2021-08-06

**Authors:** De-feng Li, Xin Chang, Jiu-long Zhao, Xuan-min Chen, Zheng-lei Xu, Ding-guo Zhang, Ben-hua Wu, Li-sheng Wang, Yu Bai, Jun Yao

**Affiliations:** ^1^Department of Gastroenterology, Shenzhen People's Hospital (The Second Clinical Medical College, Jinan University, The First Affiliated Hospital Southern University of Science and Technology), Shenzhen, 518020 Guangdong, China; ^2^Department of Gastroenterology, Changhai Hospital, Second Military Medical University, Shanghai 200082, China; ^3^Department of Gastroenterology, The General Hospital of Central Theater Command, Wuhan, 430000 Hubei, China; ^4^Department of Gastroenterology, The First Affiliated Hospital of South China University, South China University, Hengyang, 421000 Hunan, China

## Abstract

**Background:**

Ulcerative colitis (UC) is a chronic inflammatory disease with increasing prevalence worldwide. Barrier defect in intestinal epithelial cells (IECs) is one of the main pathogeneses in UC. Pyroptosis is a programmed lytic cell death and is triggered by inflammatory caspases, while little is known about its role in UC.

**Methods:**

Differentially expressed genes (DEGs) were identified by comparing UC patients with healthy controls from the GEO datasets. The candidate genes involved in pyroptosis were obtained, and the underlying molecular mechanism in the progression of UC was explored *in vivo* and *in vitro*.

**Results:**

Pleckstrin homology domain leucine-rich repeat protein phosphatase 2 (PHLPP2), a protein phosphatase, was downregulated and involved in regulating inflammation-induced IEC pyroptosis by modulating the NF-*κ*B signaling in UC through bioinformatics analysis. Moreover, we demonstrated that PHLPP2 was downregulated in UC patients and UC mice. Besides, we found that PHLPP2 depletion activated the NF-*κ*B signaling and increased the expressions of caspase-1 P20, Gasdermin N, IL-18, and IL-1*β* contributing to IEC pyroptosis and inflammation in UC mice. Furthermore, we found that PHLPP2^−/−^ mice developed hypersensitivity to dextran sulfate sodium (DSS) treatment toward colitis showing activated NF-*κ*B signaling and dramatically induced expressions of caspase-1 P20, Gasdermin N, IL-18, and IL-1*β*. Mechanically, this inflammation-induced downregulation of PHLPP2 was alleviated by an NF-*κ*B signaling inhibitor in intestinal organoids of PHLPP2^−/−^ mice and fetal colonic cells.

**Conclusions:**

PHLPP2 downexpression activated the NF-*κ*B signaling and promoted the IEC pyroptosis, leading to UC progression. Therefore, PHLPP2 might be an attractive candidate therapeutic target for UC.

## 1. Introduction

Ulcerative colitis (UC) is a chronic, idiopathic inflammatory disease with high incidence in the developed countries, and its prevalence is also increasing in the newly industrialized countries [1]. Additionally, the incidence of UC has quickly increased by nearly three times for decades in China [2]. UC is a superficial colonic mucosa inflammation starting in the rectum and extending to the proximal colon in a continuous, circumferential pattern, which is characterized by relapsing and remitting disease course with periodic flares [3].

The peak age of UC ranges from 30 to 40 years, which can lead to several symptoms, such as bloody diarrhea, abdominal discomfort, and weight loss, seriously threatening human health and affecting the quality of life [4, 5]. Although the pathogenesis of UC remains unclear, several studies have demonstrated that UC is a multifactorial disease, which is involved in genetic factors, environmental factors, dysregulated immune response, epithelial barrier defects, and inappropriate production of proinflammatory cytokines and chemokines [6–8]. Furthermore, patients with active UC are at a higher risk of colorectal cancer, especially under situations of long disease duration, primary sclerosing cholangitis (PSC), and persistent inflammation [9–11].

It is of vital importance to maintain remission and prevent complications in the long term, such as disability, colectomy, and colorectal cancer, in UC management. UC treatment is a systemic therapy, and therapeutic schemes are mainly guided by disease severity and extent. For instance, 5-aminosalicylic acid (5-ASA) is usually prescribed for mild to moderate patients, and 5-ASA suppositories can be used to treat patients with proctitis. Corticosteroids, thiopurines, or biological drugs are used in moderate to severe patients, while surgery is frequently used for uncontrolled inflammation and complications, such as perforation, intestinal obstruction, colorectal cancer, or high-grade dysplastic lesions [9].

However, UC is still a challenging medical issue, since some patients do not respond to available management [12]. Notably, the mechanisms of UC are still largely undefined [13]. In the present study, Gene Expression Omnibus (GEO) datasets showed that PHLPP2 was downregulated in the specimens of UC patients, and such downregulation promoted the pyroptosis of colonic epithelial cells, contributing to colonic inflammation. Moreover, we demonstrated that PHLPP2 was significantly decreased in the DDS-treated mice and UC patients. Furthermore, the functional loss of PHLPP2 in both mice and fetal colonic cells (FHCs) promoted the intestinal epithelial cell (IEC) pyroptosis and resulted in colon inflammatory response. We further showed that the PHLPP2 depletion directly activated NF-*κ*B signaling, promoted IEC pyroptosis, and exacerbated epithelial inflammatory response. Collectively, our findings suggested that PHLPP2 could be used as a viable therapeutic target for UC.

## 2. Materials and Methods

### 2.1. Microarray Analysis from GEO Datasets

The GSE6731, GSE11223, and GSE38713 microarray datasets were obtained from the GEO datasets, which included 87 involved active UC samples and 90 health controls. The raw data of each microarray were normalized and analyzed by R software 3.5.0 (limma and RobustRankAggreg package). Differentially expressed genes (DEGs) were screened by classical *t*-test with false discovery rate < 0.05 and ∣log FC | >1.0 as the cutoff criteria. Due to the potential functions of the DEGs, Gene Ontology (GO), Kyoto Encyclopedia of Genes and Genomes (KEGG), and Gene Set Enrichment Analysis (GSEA) were conducted by the R clusterProfiler package as previously described [14].

### 2.2. Antibodies

Antibodies against NF-*κ*B p65, p-NF-*κ*B p65, Akt, and p-Akt were purchased from Cell Signaling Technology (Boston, Massachusetts). Antibodies against GSDMD N, IKK, E-cadherin, and Iba1 antibodies were obtained from Abcam (Cambridge, MA). Antibodies against PHLPP2, caspase-1 P20, and *β*-actin were supplied by Proteintech (Rosemont, USA). Goat anti-rabbit IgG and goat anti-mouse IgG were provided by BioTNT (Shanghai, China).

### 2.3. Patients and UC Samples

Colonic tissues were obtained from active UC patients and healthy volunteers in Shenzhen People's Hospital between February 2018 and September 2019. The tissues were stored in liquid nitrogen before further use. The experimental protocols were approved by the Ethics Committee of the Shenzhen People's Hospital. This study was performed following the Helsinki Declaration. Written informed consent was obtained from all of the participants.

### 2.4. Animals

C57BL/6 mice were purchased from Shanghai Super B&K Laboratory Animal Co., Ltd. (Shanghai, China). PHLPP2^−/−^ mice were purchased from Beijing Viewsolid Biotech Co., Ltd. (Beijing, China). Mice were housed in a climate-controlled animal facility at 22°C under a 12 h light/dark cycle, and animals were given free access to water and a standard rodent diet. Mice (8~10 weeks old, weighing 20~25 g) were used in the experiment. Animal-related experiments were approved by the Animal Care Committee of the Shenzhen People's Hospital, Shenzhen, China.

### 2.5. UC Model Establishment

The mice of the UC group were administered with 4% DSS (MW 36,000 to 50,000 kDa, MP Biomedicals LLC, Santa Ana, CA, USA) dissolved in drinking water, while the control group received the drinking water without DSS. During the experimental period, the status of the body weight, diarrhea, and bleeding was observed daily. The disease activity index (DAI) was scored by the extent of body weight loss, stool hemoccult positivity or gross bleeding, and stool consistency according to the previously described method [15]. After 7 days, blood was collected through the retroorbital vein, and serum was obtained via centrifugation at 5000 × g for 15 min and stored in liquid nitrogen before further analysis. Subsequently, the animals were sacrificed, and the colons were excised. The length from the cecum to the anus was determined, and the specimens were stored in liquid nitrogen before further use.

### 2.6. Hematoxylin-Eosin (H&E) Staining and Immunofluorescence Staining

Colonic tissues were fixed with 4% paraformaldehyde overnight and then embedded in paraffin. The tissues were cut into 5 *μ*m sections, deparaffinized in xylene, washed with phosphate-buffered saline (PBS), and subjected to H&E staining.

The tissue sections were subjected to antigen retrieval in a 10 mM sodium citrate buffer containing 0.05% Tween-20 at 95°C for 30 min and then at room temperature for 20 min. The sections were washed with PBS and incubated with antibodies against PHLPP2 antibody (1 : 200) and E-cadherin (1 : 500) or Iba1 (1 : 2000) at 4°C overnight. Subsequently, the sections were washed with PBS three times and then incubated with goat anti-rabbit IgG (1 : 250) for 1 h. Images were acquired with a Nikon Eclipse 400 microscope.

### 2.7. Cells and Intestinal Organoid Culture

FHCs were purchased from the American Type Culture Collection (ATCC, Manassas, VA). FHCs were maintained in RPMI-1640 (Gibco, USA) supplemented with 10% fetal bovine serum (FBS, Gibco, USA) at 37°C in a humidified atmosphere containing 5% CO_2_. To establish a cellular model, cells were stimulated with 1 *μ*g/mL lipopolysaccharide (LPS, Sigma-Aldrich Corp) for 24 h to establish a cellular inflammatory model.

Mice were sacrificed, and the intestinal tissue was harvested. The intestine was flushed with cold PBS three times. The intestine was opened by the longitudinal incision, chopped into pieces of 2-3 mm, and then incubated in a chelating buffer (2 mM EDTA) with horizontal agitation for 30 min at 4°C to extract intestinal crypts from the basement membrane in a dissociation buffer (D-sorbitol 54.9 mM, sucrose 43.4 mM). The crypt-enriched supernatants were filtered using a cell strainer (Corning, NY, USA) four times and centrifuged at low speed (100 rpm, 3 min) 2-3 times to remove single cells. The crypts were resuspended and incubated in Tryp LE Express (Thermo Fisher Scientific) supplemented with 2,000 U/mL DNase (Roche Diagnostics, Indianapolis, IN, USA) at 37°C for 10 min. The budding number of organoids was calculated, and organoids were embedded in Matrigel for subsequent cell culture.

### 2.8. Enzyme-Linked Immunosorbent Assay (ELISA)

The levels of IL-18 and IL-1*β* in the serum and culture supernatants were quantified using commercial ELISA kits according to the manufacturer's instructions (R&D System, Minneapolis, MN, USA). The lactate dehydrogenase (LDH) levels were measured using a Pierce LDH Cytotoxicity Assay Kit (Thermo Fisher Scientific, NY, USA) following the manufacturer's instructions.

### 2.9. Cell Transfection

The transfection was performed as previously described [16]. Briefly, cells were incubated with PHLPP2-siRNA (Beijing Viewsolid Biotech, China) in the transfection medium containing Lipofectamine 2000 (Thermo Fisher Scientific, USA) at 37°C for 48 h in a humidified atmosphere containing 5% CO_2_.

### 2.10. RNA Extraction and Reverse-Transcription-Quantitative PCR (RT-qPCR)

The RNA extraction and RT-qPCR were carried out as previously described [16]. Total RNA was extracted using TRIzol (Takara, Dalian, China) and reversely transcribed into cDNA using a PrimeScript RT Master Mix according to the manufacturer's instructions (Takara, Dalian, China). RT-qPCR was performed on a 7500 Real-Time PCR System with SY FastStart Universal SYBR Green Master (Roche, Basel, Switzerland) according to the manufacturer's instructions. The following primers were used: PHLPP2: forward, 5′-CCTCCTCCTCCTCCTCCTCCTC-3′, and reverse, 5′-GGCTCCAGTCTCCTGACCAGATC-3′; GAPDH: forward, 5′-GTCTCCTCTGACTTC AACAGCG-3′, and reverse, 5′-ACCACCCTGTTGCTGTAG CCAA-3′. GAPDH was adopted as a housekeeping gene. Briefly, after an initial denaturation step at 95°C for 60 s, the amplifications were carried out with 40 cycles at a melting temperature of 95°C for 15 s and an annealing temperature of 60°C for 30 s. The relative expressions of the target genes were calculated using the 2^-△△Ct^ method.

### 2.11. Western Blotting

Total proteins from the colonic tissues and cells were extracted using a standard extraction reagent supplemented with the protease inhibitor (KANGCHEN; Shanghai, China). Protein concentrations were calculated through the BCA kit (Beyotime, China), according to the manufacturer's instructions. Equal amounts of proteins (30 *μ*g) were subjected to the SDS-PAGE gel and then transferred onto a polyvinylidene fluoride (PVDF; Millipore, USA) membrane. The membranes were blocked with 5% skim milk in PBS at room temperature for 1 h, followed by the incubation with the primary antibodies against rabbit anti-Akt (1 : 1,000), rabbit anti-p-Akt (1 : 2,000), rabbit anti-IKK (1 : 1,000), rabbit anti-NF-*κ*B p65 (1 : 1,000), rabbit anti-p-NF-*κ*B p65 (1 : 1,000), rabbit anti-caspase-1 P20 (1 : 1,000), rabbit anti-Gasdermin N (1 : 1,000), rabbit anti-PHLPP2 (1 : 2,000), and *β*-actin (1 : 2,000) for 24 h at 4°C. Subsequently, the blots were washed with TBST three times (10 min for each wash) and incubated with anti-rabbit IgG (1 : 10,000) for 1 h. Finally, immunoreactive bands were visualized with an Odyssey infrared scanning system (LI-COR Biosciences, USA) and quantified using ImageJ software.

### 2.12. Statistical Analysis

Data were expressed as means ± standard error of the means (SEM). Differences between the two groups were analyzed using a two-way analysis of variance (ANOVA) followed by Bonferroni's post hoc test. A Kruskal–Wallis test and one-way ANOVA were used to assess nonparametric data and continuous variables, respectively. All analyses were performed using the SPSS 23.0 software package (SPSS Company, Chicago, IL, USA). *P* values < 0.05 were considered statistically significant.

## 3. Results

### 3.1. PHLPP2 Is Downregulated and Regulates Pyroptosis in UC by Bioinformatics Analysis

**Figure 1:**
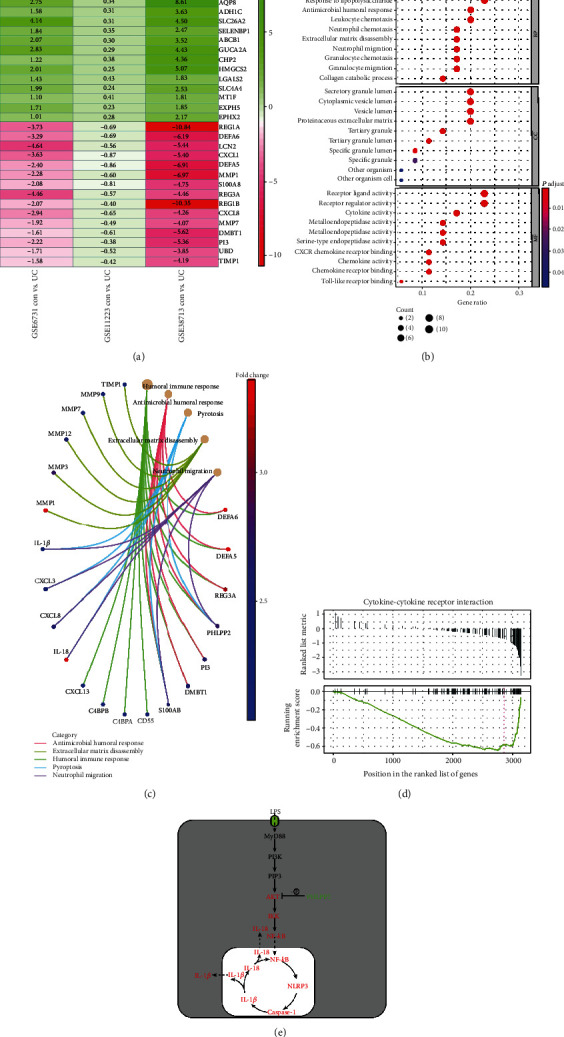
The DEGs from GEO datasets. (a) The DEGs were obtained from GSE6731, GSE11223, and GSE38731 (∣log FC | >1.0, *P* < 0.05). The top 15 DEGs were shown in the figure, and red/blue are relatively upregulated/downregulated by comparing active UC patients with healthy controls. (b) GO analysis showed that the DEGs were especially involved in humoral immune response, pyroptosis, and neutrophile migration. (c) KEGG pathway analysis showed that the DEGs were associated with pyroptosis. (d) GSEA analysis showed that the DEGs were enriched by cytokine-cytokine receptor interaction. (e) Bioinformatics analysis showed downexpression of the PHLPP2-activated NF-*κ*B signaling pathway and contributed to pyroptosis in UC.

The pathogenesis of UC is involved in genetic predisposition [17]. Therefore, we performed available whole-genome transcriptional analysis from the GEO dataset. We found that there were significant differences between involved mucosa from patients with active UC and healthy controls from GSE6731, GSE11223, and GSE38713 (Figure 1(a)). Table S1 shows that there were 41 upregulated genes and 16 downregulated genes in the UC patients compared with the healthy controls (|log FC| >1.0, *P* < 0.05). Indeed, GO analysis revealed that the DEGs were specifically associated with humoral immune response and neutrophil migration (Figure 1(b)). KEGG analysis demonstrated that DEGs were involved in pyroptosis (Figure 1(c)). Moreover, GSEA implied that DEGs were significantly enriched in cytokine-cytokine receptor interaction (Figure 1(d)) within inflamed mucosa. Collectively, bioinformatics analysis demonstrated that the downexpression of PHLPP2 might regulate the pyroptosis of colonic epithelial cells via activating the NF-*κ*B signaling in UC (Figure 1(e)).

### 3.2. PHLPP2 Is Downregulated in the Inflamed Mucosa of UC

**Figure 2:**
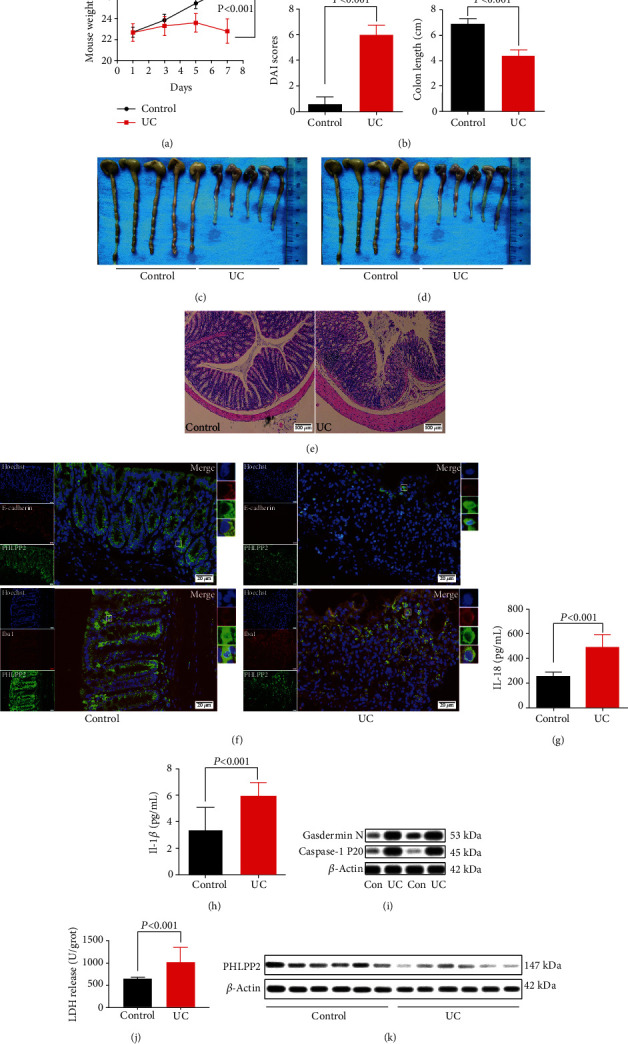
PHLPP2 is downregulated and contributes to pyroptosis in UC. (a–d) Compared with the control group, the UC group exhibited significant weight loss, increased DAI, and shortened colon length. (e) H&E staining of colon sections showed aggravated inflammatory infiltration in the UC group (100x). (f) Coimmunofluorescence staining of PHLPP2 and E-cadherin or Iba1 showed that PHLPP2 was located in IECs and downregulated in the UC group. (g, h) The UC group had significantly enhanced IL-18 and IL-1*β* levels. (i) The levels of caspase-1 P20 and Gasdermin N were significantly elevated in the colons of the UC group. (j) LDH activity was significantly increased in the colons of the UC group. (k) PHLPP2 was significantly decreased in the colons of the UC patients.

It is well known that oral administration of DSS induces intestinal inflammation which is clinically and histologically reminiscent of human UC [18]. Therefore, the C57 mice were administrated 4% DSS for 7 days in the drinking water to induce acute colitis [19]. The weight loss and DAI were significantly greater in the UC group compared with the control group (Figures 2(a) and 2(b)). Moreover, the colons of the UC group were significantly shorter compared with those of the control group (Figures 2(c) and 2(d)). Besides, H&E staining showed that inflammation and tissue damage were distinctly observed in the colon of the UC group (Figure 2(e)). Furthermore, coimmunofluorescence staining of PHLPP2 and E-cadherin or Iba1 demonstrated that PHLPP2 was predominantly observed in IECs, whereas the proportion of PHLPP2-positive cells was significantly decreased in the UC group (Figure 2(f)).

It has been reported that DSS-induced colitis can promote the release of proinflammatory mediators [13].Therefore, the serum levels of IL-18 and IL-1*β* were detected by ELISA, which were prominently increased in the UC group compared with the control group (Figures 2(g) and 2(h)).

As a programmed lytic cell death, pyroptosis is associated with a highly inflammatory response, which is distinct from apoptosis and necrosis [20]. It has been reported that inflammasomes activate caspase-1 P20, and then, caspase-1 P20 cleaves Gasdermin D to Gasdermin N and induces pyroptosis, subsequently leading to secretion of IL-18 and IL-1*β* [21]. Consistent with IL-18 and IL-1*β*, the expressions of caspase-1 P20 and Gasdermin N were significantly elevated in the colons of the UC group compared with the control group (Figure 2(i)). Moreover, it has been reported that LPS promotes lung endothelial pyroptosis and induces LDH release [22]. Therefore, we examined the LDH activity and found that the LDH activity was significantly greater in the colons of the UC group compared with the control group (Figure 2(j)).

Besides, we found that PHLPP2 was dramatically downregulated in the human samples from 42 active UC patients compared with 24 healthy controls (Figure 2(k)). Taken together, these findings indicated that the downexpression of PHLPP2 might promote IEC pyroptosis and contribute to epithelial inflammation.

### 3.3. PHLPP2 Depletion Promotes Colonic Epithelial Pyroptosis and Exacerbates the Inflammatory Response by Activating the NF-*κ*B Signaling *In Vivo*

**Figure 3:**
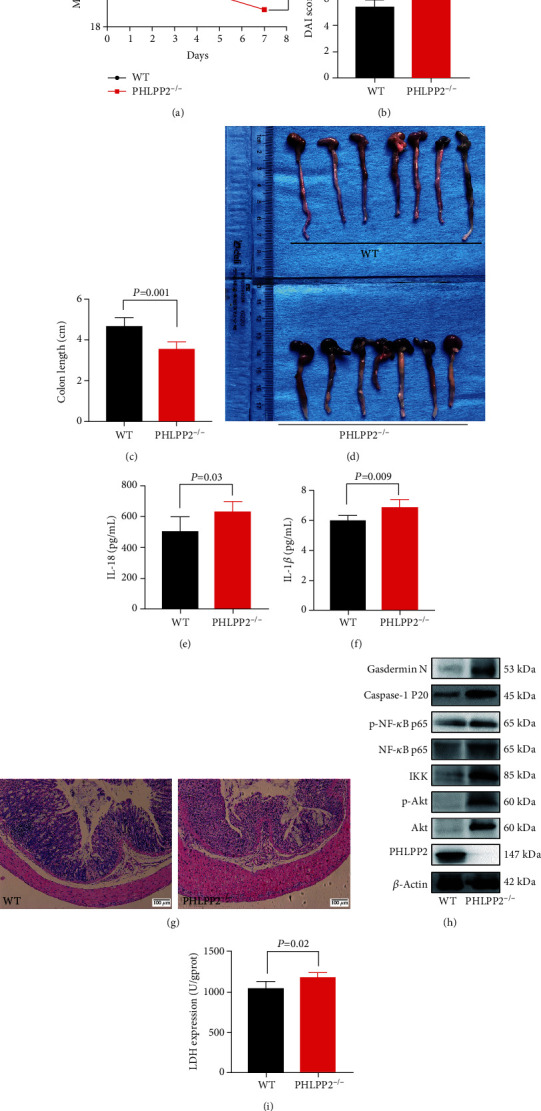
PHLPP2 depletion exacerbates the DSS-induced colitis. (a–d) Compared with the WT group, the PHLPP2^−/−^ group exhibited significant weight loss, increased DAI, and shortened colon length after DSS administration. (e, f) The PHLPP2^−/−^ group had significantly enhanced IL-18 and IL-1*β* levels compared with the WT group after DSS administration. (g) H&E staining of colon sections showed aggravated inflammatory infiltration in the PHLPP2^−/−^ group (100x). (h) PHLPP2 was significantly decreased; however, the expressions of p-Akt, IKK, NF-*κ*B p65, p-NF-*κ*B p65, caspase-1 P20, and Gasdermin N dramatically increased in the PHLPP2^−/−^ group compared with the WT group after DSS administration. (i) LDH activity was significantly upregulated in the colons of the PHLPP2^−/−^ group compared with the WT group after DSS administration.

**Figure 4:**
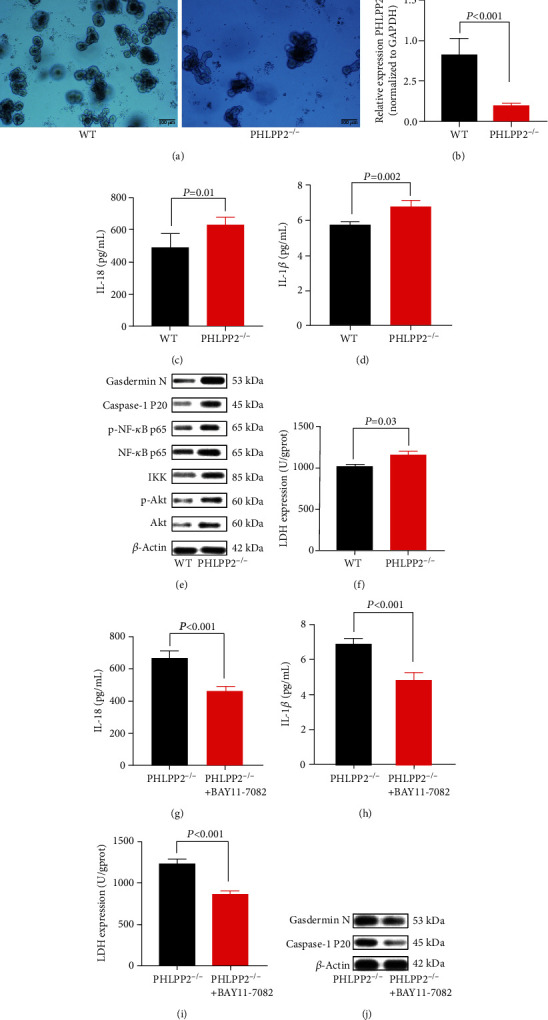
PHLPP depletion contributes to colonic organoid pyroptosis after LPS exposure. (a) Colonic organoids were cultured for 4 days in the WT group and PHLPP2^−/−^ group. (b) PHLPP2 was significantly decreased in the PHLPP2^−/−^ group. (c, d) The PHLPP2^−/−^ group had significantly enhanced IL-18 and IL-1*β* levels after LPS exposure. (e) The PHLPP2^−/−^ group had significantly increased the expressions of p-Akt, IKK, NF-*κ*B p65, p-NF-*κ*B p65, caspase-1 P20, and Gasdermin N after LPS exposure. (f) LDH activity was significantly upregulated in the colons of the PHLPP2^−/−^ group after LPS was induced. (g–i) The NF-*κ*B signaling inhibitor can alleviate the inflammation and significantly decrease IL-18, IL-1*β*, and LDH levels in the PHLPP2^−/−^ group. (j) The NF-*κ*B signaling inhibitor can significantly decrease the expression of caspase-1 P20 and Gasdermin N expression in the PHLPP2^−/−^ group.

To explore the effect of PHLPP2 deletion on the colon, we established a PHLPP2^−/−^ mouse model. PHLPP2^−/−^ and WT mice were subjected to 4% DSS treatment for 7 days. Compared with the WT mice, the PHLPP2^−/−^ mice exhibited a significantly higher sensitivity to DSS exposure, with a greater weight loss and DAI (Figures 3(a) and 3(b)). As expected, DSS-treated PHLPP2^−/−^ mice had a shorter colon length (Figures 3(c) and 3(d)). Besides, DSS administration induced a significantly greater secretion of IL-18 and IL-1*β* in the PHLPP2^−/−^ mice (Figures 4(e) and 3(f)). Furthermore, tissue damage, crypt loss, and inflammatory cell infiltration were significantly observed in the PHLPP2^−/−^ mice (Figure 3(g)). Indeed, Western blotting analysis demonstrated that the expression of PHLPP2 was markedly lower in the PHLPP2^−/−^ mice. In contrast, the expressions of Akt, p-Akt, IKK, NF-*κ*B p65, p-NF-*κ*B p65, caspase-1 P20, and Gasdermin N were significantly increased in the PHLPP2^−/−^ mice (Figure 3(h)). Moreover, the activity of LDH was significantly higher in the PHLPP2^−/−^ mice (Figure 3(i)). Therefore, PHLPP2^−/−^ mice developed hypersensitivity to DSS treatment toward colitis, which might be mainly attributed to NF-*κ*B signaling activation and the subsequent induction of IEC pyroptosis.

### 3.4. PHLPP2 Depletion Promotes Colonic Epithelial Pyroptosis by Activating the NF-*κ*B Signaling *In Vitro*

To further investigate the molecular mechanisms underlying the regulatory effects of PHLPP2 on IEC pyroptosis, we obtained intestinal organoids initiated from a single perforating cell in PHLPP2^−/−^ and WT mice (Figure 4(a)). It was shown that the PHLPP2 expression was significantly lower in the PHLPP2^−/−^ group compared with the WT group (Figure 4(b)). After organoids were treated with LPS for 24 h, the expressions of IL-18 and IL-1*β* were significantly upregulated in the PHLPP2^−/−^ group compared with the WT group (Figures 4(c) and 4(d)). Consistent with the *in vivo* findings, the expressions of Akt, p-Akt, IKK, NF-*κ*B p65, p-NF-*κ*B p65, caspase-1 P20, and Gasdermin N were dramatically upregulated (Figure 4(e)), and the LDH activity was significantly increased in the PHLPP2^−/−^ group after LPS treatment (Figure 4(f)). However, BAY11-7082, an NF-*κ*B signaling inhibitor, alleviated the inflammation and significantly decreased the expressions of IL-18, IL-1*β*, and LDH (Figures 4(g)–4(i)), as well as the expressions of caspase-1 P20 and Gasdermin N in the PHLPP2^−/−^ group (Figure 4(j)). Collectively, these results demonstrated that PHLPP2 depletion promoted the colonic epithelial pyroptosis and contributed to the inflammatory response by activating the NF-*κ*B signaling pathway.

**Figure 5:**
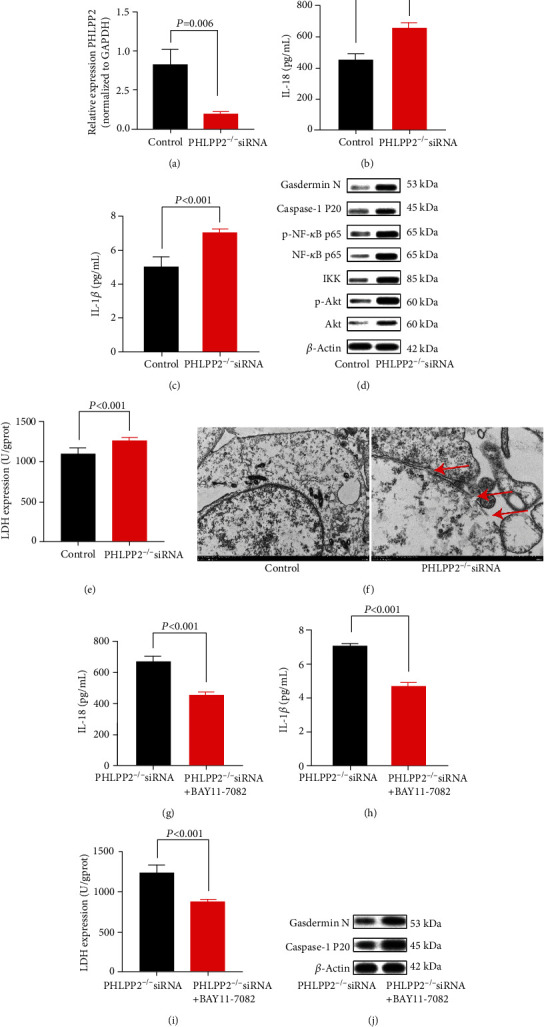
PHLPP depletion contributes to FHC pyroptosis after LPS exposure. (a) The PHLPP2 was significantly decreased in the PHLPP2-siRNA group. (b, c) Compared with the control group, the PHLPP2-siRNA group had significantly enhanced IL-18 and IL-1*β* levels after LPS exposure. (d) The PHLPP2-siRNA group had significantly increased the expression of p-Akt, IKK, NF-*κ*B p65, p-NF-*κ*B p65, caspase-1 P20, and Gasdermin N after LPS exposure. (e) LDH activity was significantly increased in the colons of the PHLPP2-siRNA group after LPS exposure. (f) Pyroptosis was detected using scanning electron microscopy (SEM). Typical membrane integrity loss was found in the PHLPP2-siRNA group. (g–i) The NF-*κ*B signaling inhibitor can alleviate the inflammation and significantly decrease IL-18, IL-1*β*, and LDH levels in the PHLPP2-siRNA group. (j) The NF-*κ*B signaling inhibitor can significantly decrease caspase-1 P20 and Gasdermin N expressions in the PHLPP2-siRNA group.

Next, we silenced of PHLPP2 in FHCs and confirmed that the PHLPP2 expression was significantly decreased in the PHLPP2-siRNA group compared with the control group (Figure 5(a)). After treating with LPS for 24 h, the expressions of IL-18 and IL-1*β* were significantly increased in the PHLPP2-siRNA group compared with the control group (Figures 5(b) and 5(c)). Consistently, the expressions of Akt, p-Akt, IKK, NF-*κ*B p65, p-NF-*κ*B p65, caspase-1 P20, and Gasdermin N were significantly upregulated, and the LDH activity was significantly elevated in the PHLPP2-siRNA group (Figures 5(d) and 5(e)). Besides, scanning electron microscopy (SEM) membrane indicated that FHCs lost membrane integrity and underwent pyroptosis in the PHLPP2-siRNA group (Figure 5(f)). However, the NF-*κ*B inhibitor, BAY11-7082, also alleviated the inflammation and decreased the expressions of IL-18, IL-1*β*, and LDH expression (Figures 5(g)–5(i)), and the expressions of caspase-1 P20 and Gasdermin N were also downregulated in the PHLPP2-siRNA group (Figure 5(j)). Therefore, these results indicated that PHLPP2 depletion promoted the colonic epithelial pyroptosis and contributed to the inflammatory response by activating the NF-*κ*B signaling.

## 4. Discussion

As a general innate immune mechanism, pyroptosis is characterized by cell swelling, lysis, and release of proinflammatory cytokines and intracellular content via the canonical pathway and noncanonical pathway [23]. Extensive studies have demonstrated that in the canonical pathway, caspases-1 P20 cleaves Gasdermin D to Gasdermin N, and extensive pores form on the cell plasma membrane, leading to cell swelling and eventual lysis [24–26]. A previous study has indicated that caspase-1 P20 exclusively cleaves Gasdermin D to Gasdermin N and mediates pyroptosis in mouse macrophages [27, 28], while Gasdermin D is widely expressed in different cell types and tissues, especially with high expressions in the gastrointestinal epithelium [29], suggesting that pyroptosis is not limited to mouse macrophages. In this present study, we first demonstrated that the expressions of caspases-1 P20 and Gasdermin N were significantly elevated in DSS-fed mice, and the expressions of IL-18, IL-1*β*, and LDH were significantly increased, supporting that pyroptosis might occur in UC.

PHLPP2 and its related paralog PHLPP1 are members of the PHLPP family, which can inactivate signaling of their targets Akt and PKC by dephosphorylation [30]. Indeed, PHLPP2 plays a crucial role in various cellular processes, such as suppression of bladder cancer [31], inhibition of hepatic steatosis [32], and induction of IEC apoptosis [33]. In the present study, we demonstrated that PHLPP2 was located in the IECs, and its expression was significantly decreased in DSS-fed mice as well as human tissues of UC patients. Indeed, more weight loss, less colon length and higher DAI, and greater expressions of caspases-1 P20, Gasdermin N, IL-18, IL-1*β*, and LDH were found in the PHLPP2^−/−^ mice compared with the WT mice after DSS administration. These findings indicated that PHLPP2 deficiency exacerbated colitis in PHLPP2^−/−^ mice. Furthermore, the expressions of caspase-1 P20, Gasdermin N, IL-18, IL-1*β*, and LDH were significantly increased in the organoids from colonic epithelia of PHLPP2^−/−^ mice compared with the WT mice treated with 4% DSS. Therefore, it suggested that PHLPP2 depletion led to colonic epithelial pyroptosis and promoted colitis progression. Nevertheless, Wen et al. have demonstrated that depletion of PHLPP2 activates the NF-*κ*B signaling pathway and inhibits IEC apoptosis [33].

**Figure 6:**
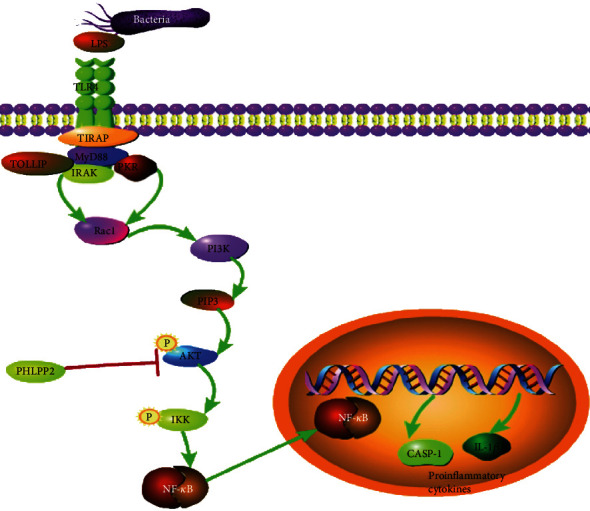
Schematic illustration. PHLPP2 depletion activates NF-*κ*B signaling activation, induces IEC pyroptosis, and exacerbates colonic inflammation in UC.

It is well known that the signaling pathway engages in pyroptosis, which promotes the release of proinflammatory cytokines and chemokines within the cells and induces inflammation [25, 34]. In this study, we found that downexpression of PHLPP2 increased the activation of the NF-*κ*B signaling pathway; upregulated the expressions of Akt, p-Akt, IKK, NF-*κ*B p65, p-NF-*κ*B p65, caspase-1 P20, Gasdermin N, IL-18, IL-1*β*, and LDH; promoted IEC pyroptosis; and exacerbated colonic inflammatory response. Nevertheless, pyroptosis could be inhibited and inflammation could be alleviated by an NF-*κ*B inhibitor. Therefore, these findings supported that the downexpression of PHLPP2 promoted the IEC pyroptosis and exacerbated the colonic inflammatory response by activating NF-*κ*B signaling. Interestingly, a recent study has shown that the activation of NF-*κ*B signaling can induce tubular cell pyroptosis and contribute to the progression of tubular injury in kidney disease, which is consistent with our results [35]. Furthermore, it has been discovered that inhibition of NF-*κ*B signaling suppresses the mucosal inflammation in IBD [36]. Additional studies have shown that IKKs, including IkB kinases a and b, are the major protein kinases to activate NF-*κ*B complexes, as well as the IKK kinase complex, which is the key point in the NF-*κ*B-mediated inflammatory signaling [37–39]. Moreover, Akt kinase can activate NF-*κ*B signaling via IKK phosphorylation [40, 41]. However, it has been demonstrated that PHLPP2 inactivates Akt via dephosphorylation, resulting in inhibited NF-*κ*B signaling [30, 42]. Therefore, we considered that the downexpression of PHLPP2 activated NF-*κ*B signaling activation, induced the IEC pyroptosis, and exacerbated the colonic inflammation in UC (Figure 6).

In conclusion, we, for the first time, showed that downexpression of PHLPP2 directly activated the NF-*κ*B signaling and promoted the IEC pyroptosis, leading to the progression of colitis in UC. Given our findings, PHLPP2 might be an attractive candidate therapeutic target for UC.

## Data Availability

All data generated or analyzed during this study are available from the corresponding author Jun Yao upon reasonable request.

## Supplementary Materials

Supplementary Materials Table S1: the different expression genes from GEO datasets.

## References

[1] Kaser A., Zeissig S., Blumberg R. S. (2010). Inflammatory bowel disease. *Annual Review of Immunology*.

[2] Ye L., Cao Q., Cheng J. (2013). Review of inflammatory bowel disease in China. *Scientific World Journal*.

[3] Kim D. H., Cheon J. H. (2017). Pathogenesis of inflammatory bowel disease and recent advances in biologic therapies. *Immune network*.

[4] Høivik M. L., Moum B., Solberg I. C. (2013). Work disability in inflammatory bowel disease patients 10 years after disease onset: results from the IBSEN study. *Gut*.

[5] Cosnes J., Gower–Rousseau C., Seksik P., Cortot A. (2011). Epidemiology and natural history of inflammatory bowel diseases. *Gastroenterology*.

[6] Neurath M. F. (2014). Cytokines in inflammatory bowel disease. *Nature Reviews. Immunology*.

[7] Ng S. C., Shi H. Y., Hamidi N. (2017). Worldwide incidence and prevalence of inflammatory bowel disease in the 21st century: a systematic review of population-based studies. *Lancet*.

[8] Swiss IBD Cohort Investigators, Yilmaz B., Juillerat P. (2019). Microbial network disturbances in relapsing refractory Crohn's disease. *Nature Medicine*.

[9] Ungaro R., Mehandru S., Allen P. B., Peyrin-Biroulet L., Colombel J. F. (2017). Ulcerative colitis. *Lancet*.

[10] Ford A. C., Moayyedi P., Hanauer S. B. (2013). Ulcerative colitis. *BMJ*.

[11] Feuerstein J. D., Cheifetz A. S. (2014). Ulcerative colitis: epidemiology, diagnosis, and management. *Mayo Clinic Proceedings*.

[12] Abdulrazeg O., Li B., Epstein J. (2019). Management of ulcerative colitis: summary of updated NICE guidance. *BMJ*.

[13] Neurath M. F. (2019). Targeting immune cell circuits and trafficking in inflammatory bowel disease. *Nature Immunology*.

[14] Chang X., Yang M. F., Fan W. (2020). Bioinformatic analysis suggests that three hub genes may be a vital prognostic biomarker in pancreatic ductal adenocarcinoma. *Journal of Computational Biology*.

[15] Wang J., Zhu G., Sun C. (2020). TAK-242 ameliorates DSS-induced colitis by regulating the gut microbiota and the JAK2/STAT3 signaling pathway. *Microbial Cell Factories*.

[16] Li D., Yang M., Liao A. (2018). Linc00483 as ceRNA regulates proliferation and apoptosis through activating MAPKs in gastric cancer. *Journal of Cellular and Molecular Medicine*.

[17] Planell N., Lozano J. J., Mora-Buch R. (2013). Transcriptional analysis of the intestinal mucosa of patients with ulcerative colitis in remission reveals lasting epithelial cell alterations. *Gut*.

[18] Kitajima S., Takuma S., Morimoto M. (1999). Changes in colonic mucosal permeability in mouse colitis induced with dextran sulfate sodium. *Experimental Animals*.

[19] Demon D., Kuchmiy A., Fossoul A., Zhu Q., Kanneganti T. D., Lamkanfi M. (2014). Caspase-11 is expressed in the colonic mucosa and protects against dextran sodium sulfate-induced colitis. *Mucosal Immunology*.

[20] Jorgensen I., Rayamajhi M., Miao E. A. (2017). Programmed cell death as a defence against infection. *Nature Reviews. Immunology*.

[21] Broz P., Dixit V. M. (2016). Inflammasomes: mechanism of assembly, regulation and signalling. *Nature Reviews. Immunology*.

[22] Cheng K. T., Xiong S., Ye Z. (2017). Caspase-11-mediated endothelial pyroptosis underlies endotoxemia-induced lung injury. *The Journal of Clinical Investigation*.

[23] Fink S. L., Cookson B. T. (2006). Caspase-1-dependent pore formation during pyroptosis leads to osmotic lysis of infected host macrophages. *Cellular Microbiology*.

[24] Shi J., Gao W., Shao F. (2017). Pyroptosis: gasdermin-mediated programmed necrotic cell death. *Trends in Biochemical Sciences*.

[25] Green D. R. (2019). The coming decade of cell death research: five riddles. *Cell*.

[26] Yuan J., Najafov A., Py B. F. (2016). Roles of caspases in necrotic cell death. *Cell*.

[27] Kayagaki N., Stowe I. B., Lee B. L. (2015). Caspase-11 cleaves gasdermin D for non-canonical inflammasome signalling. *Nature*.

[28] Shi J., Zhao Y., Wang K. (2015). Cleavage of GSDMD by inflammatory caspases determines pyroptotic cell death. *Nature*.

[29] Saeki N., Kuwahara Y., Sasaki H., Satoh H., Shiroishi T. (2000). Gasdermin (Gsdm) localizing to mouse chromosome 11 is predominantly expressed in upper gastrointestinal tract but significantly suppressed in human gastric cancer cells. *Mammalian Genome*.

[30] Brognard J., Sierecki E., Gao T., Newton A. C. (2007). PHLPP and a second isoform, PHLPP2, differentially attenuate the amplitude of Akt signaling by regulating distinct Akt isoforms. *Molecular Cell*.

[31] Peng M., Wang J., Zhang D. (2018). PHLPP2 stabilization by p27 mediates its inhibition of bladder cancer invasion by promoting autophagic degradation of MMP2 protein. *Oncogene*.

[32] Kim K., Ryu D., Dongiovanni P. (2017). Degradation of PHLPP2 by KCTD17, via a Glucagon-Dependent Pathway, Promotes Hepatic Steatosis. *Gastroenterology*.

[33] Wen Y. A., Li X., Goretsky T., Weiss H. L., Barrett T. A., Gao T. (2015). Loss of PHLPP protects against colitis by inhibiting intestinal epithelial cell apoptosis. *Biochimica et Biophysica Acta (BBA)-Molecular Basis of Disease*.

[34] Jia C., Zhang J., Chen H. (2019). Endothelial cell pyroptosis plays an important role in Kawasaki disease via HMGB1/RAGE/cathespin B signaling pathway and NLRP3 inflammasome activation. *Cell Death & Disease*.

[35] Wang Y., Zhu X., Yuan S. (2019). TLR4/NF-*κ*B signaling induces GSDMD-related pyroptosis in tubular cells in diabetic kidney disease. *Frontiers in endocrinology*.

[36] du X., Chen W., Wang Y. (2017). Therapeutic efficacy of carboxyamidotriazole on 2,4,6-trinitrobenzene sulfonic acid-induced colitis model is associated with the inhibition of NLRP3 inflammasome and NF-*κ*B activation. *International Immunopharmacology*.

[37] Gloire G., Dejardin E., Piette J. (2006). Extending the nuclear roles of I*κ*B kinase subunits. *Biochemical Pharmacology*.

[38] Perkins N. D. (2007). Integrating cell-signalling pathways with NF-*κ*B and IKK function. *Nature Reviews. Molecular Cell Biology*.

[39] Salminen A., Huuskonen J., Ojala J., Kauppinen A., Kaarniranta K., Suuronen T. (2008). Activation of innate immunity system during aging: NF-kB signaling is the molecular culprit of inflamm-aging. *Ageing Research Reviews*.

[40] Cho J. A., Kim T. J., Moon H. J., Kim Y. J., Yoon H. K., Seong S. Y. (2018). Cardiolipin activates antigen-presenting cells via TLR2-PI3K-PKN1-AKT/p38-NF-kB signaling to prime antigen-specific naïve T cells in mice. *European Journal of Immunology*.

[41] Han M. H., Lee W. S., Nagappan A. (2016). Flavonoids isolated from flowers ofLonicera japonicaThunb. inhibit inflammatory responses in BV2 microglial cells by suppressing TNF-*α* and IL-*β* through PI3K/Akt/NF-kb signaling pathways. *Phytotherapy Research*.

[42] Toivanen R., Furic L. (2019). A balancing act: PHLPP2 fine tunes AKT activity and MYC stability in prostate cancer. *The Journal of Cell Biology*.

