# Supplementary material for: Colonic Epithelial PHLPP2 Deficiency Promotes Colonic Epithelial Pyroptosis by Activating the NF-κB Signaling Pathway
Source: Oxid Med Cell Longev. 2021 Aug 6;2021:5570731. doi: 10.1155/2021/5570731 (PMC8363454; doi:10.1155/2021/5570731)
Supplement: Supplementary Materials — Table S1: the different expression genes from GEO datasets. [file 5570731.f1.docx]

Table S1 The different expression genes from GEO datasets.

|  | Genes | | | |
| --- | --- | --- | --- | --- |
|  | REG1A | DEFA6 | LCN2 | CXCL1 |
|  | S100A9 | CXCL3 | PLAU | IL1B |
|  | CXCL8 | MMP7 | DMBT1 | PI3 |
|  | CD55 | SELL | C4BPB | TCN1 |
|  | MMP1 | S100A8 | REG3A | REG1B |
| Up-expression | TIMP1 | OLFM4 | CASP1 | CFI |
|  | ISG20 | IFITM3 | IDO1 | NOS2 |
|  | BCL2A1 | PLEK | TGFBI | TMEM158 |
|  | MMP12 | DEFA5 | C4BPA | UBD |
|  | SPINK4 | CXCL13 | S100P | MMP9 |
|  | MMP3 |  |  |  |
|  | CLDN8 | PCK1 | AQP8 | ADH1C |
|  | HMGCS2 | SLC4A4 | MT1F | EXPH5 |
| Down-expression | SELENBP1 | ABCB1 | GUCA2A | CHP2 |
|  | PHLPP2 | SLC26A2 | EPHX2 | TSPAN7 |
